# New Observations upon the Etiology of Eclampsia

**Published:** 1886-08-20

**Authors:** 


**New Observations upon the Etiology of Eclampsia.** — At a meeting of the Societe de Biologie (Le Progres Medical) MM. Doleris and Butte communicated the results of some chemical and experimental observations upon eclampsia, made upon five patients. In the first examined the authors found, in the ethereal extract of the blood treated with dilute acid, a crystalline substance, which was inorganic, or at least partly so, soluble in ether and in acidulated water, insoluble in water, very slightly soluble in alcohol, and which did not behave like a tromaine as regards certain color-reactions. These crystals are toxic, and may kill rats and sparrows rather quickly, even in almost infinitesimal doses ; the symptoms being first a stage of excitation, then a period of somnolence, followed by death in five or six hours. In the fifth case of eclampsia the crystals were not detected. In the first four the proportion of urea in the blood was not excessive, and was, moreover, considerably lower than in the last one, in which no crystals were found. This analysis indicated that the accumulation of urea in the blood is too slight to enable us to attribute to it a *role* in the pathogeny of eclampsia.—*Philadelphia Med. Times.*

